# Association of dietary phytochemical index with metabolically unhealthy overweight/obesity phenotype among Iranian women: A cross-sectional study

**DOI:** 10.3389/fnut.2022.959341

**Published:** 2022-10-25

**Authors:** Sanaz Pourreza, Atieh Mirzababaei, Fatemeh Naeini, Sina Naghshi, Khadijeh Mirzaei

**Affiliations:** ^1^Department of Community Nutrition, School of Nutritional Sciences and Dietetics, Tehran University of Medical Sciences (TUMS), Tehran, Iran; ^2^Department of Clinical Nutrition, School of Nutritional Sciences and Dietetics, Tehran University of Medical Sciences, Tehran, Iran

**Keywords:** phytochemical index, obesity, metabolically healthy, obesity phenotypes, overweight

## Abstract

**Background:**

Phytochemicals have been recently studied as adjuvants for the treatment of obesity. No study has investigated the association of phytochemical-rich foods with metabolically unhealthy overweight/obesity phenotype (MUOW/O). This study aimed to determine the association of dietary phytochemical index (DPI) with MUOW/O based on Karelis criteria among Iranian female adults.

**Methods:**

In this cross-sectional study, a total of 228 overweight and obese women aged 18–48 years were included. Anthropometric measurements were evaluated for all participants. A validated 147-item Food Frequency Questionnaire (FFQ) was used for dietary assessment. DPI was calculated as [dietary energy derived from phytochemical-rich foods (kcal)/total daily energy intake (kcal)] × 100. Participants’ body composition and biochemical parameters of Karelis criteria [triglyceride (TG), high-density lipoprotein cholesterol (HDL-C), low-density lipoprotein cholesterol (LDL-C), insulin, and high-sensitivity C-reactive protein (hs-CRP)] were determined.

**Results:**

The mean age of the study participants was 36.69 ± 9.20, and the mean DPI score was 26.23 ± 9.48 among participants with MUOW/O phenotype. After controlling for potential confounders, women in the highest tertile of DPI had lower odds for MUOW/O phenotype [odds ratio (OR): 0.23, 95% confidence interval (CI): 0.07–0.68, *P* = 0.008] compared to the lowest tertile. Among the components of Karelis criteria, homeostatic model assessment for insulin resistance (HOMA-IR) was significantly associated with MUOW/O phenotype in the fully adjusted model (OR: 0.29, 95% CI: 0.10–0.79, *P* = 0.01).

**Conclusion:**

We found a significant association between DPI and MUOW/O phenotype in Iranian women. Prospective studies are needed to confirm these findings.

## Background

Obesity is a prevalent disease that is described as an epidemic with its globally increasing high rate (1). It is estimated that almost 2.1 billion people are either overweight or obese around the world (2). Obesity is caused by a constant positive energy balance, which may stem from various genetic, social, and personal factors (2, 3). Obesity is strongly linked to multiple metabolic disorders, including diabetes mellitus (DM), cardiovascular diseases (CVDs), hypertension, dyslipidemia, and inflammation-related conditions (4, 5).

Despite these facts, not all obese people develop metabolic dysfunction (6). It has been recognized that some obese individuals have a favorable metabolic profile, including blood pressure, lipid and hormonal profile, insulin sensitivity, and a lower risk of CVDs (7, 8). This subcategory of obesity is defined as metabolically healthy obesity (MHO) phenotype (7). The prevalence of MHO is controversial; however, based on different criteria and populations, it is estimated to be 6–75% (9, 10). This phenotype results from a complex interaction of multiple factors, e.g., genetics, environment, lifestyle, and diet (6, 11, 12). On the contrary, the metabolically unhealthy obesity (MUO) phenotype is connected with at least two or more metabolic disorders and more susceptibility to CVDs (13). A 10-year follow-up demonstrated that almost half the people with MHO phenotype would develop one or more metabolic abnormalities (14). Thus, MHO is a rather transient phenotype (10, 14, 15). Several criteria have been suggested to define MHO phenotypes (16, 17). Karelis criteria (18) measure triglyceride (TG), low-density lipoprotein cholesterol (LDL-C), high-density lipoprotein cholesterol (HDL-C), high-sensitivity C-reactive protein (hs-CRP), and homeostatic model assessment for insulin resistance (HOMA-IR) to determine MHO. In the current study, Karelis criteria have been used since it considers both insulin resistance and inflammation as practical measurements of health evaluation in the obese population (18) and demands at least four of the proposed criteria to introduce an individual as having MHO phenotype (19).

A significant factor that affects the metabolic health of obese individuals is diet (20). Among dietary factors, foods rich in phytochemical compounds such as fruits, vegetables, and whole grains are associated with weight control and the prevention of chronic and metabolic diseases, e.g., CVDs, type 2 DM, and metabolic syndrome (21). Phytochemicals, also called natural secondary plant metabolites, include a large group of compounds, such as phenolic compounds, organosulfur compounds, flavonoids, carotenoids, and alkaloids (22–24). It has been shown that phytochemicals regulate carbohydrate and lipid metabolism and have anti-inflammatory and anti-oxidative characteristics (25). Since it is quite troublesome to determine the amount of phytochemical intake in large populations (26), McCarty (27) proposed a practical tool, the phytochemical index (PI), which is defined as the percentage of calorie intake derived from phytochemical-rich foods. Previous studies have assessed the association between PI and metabolic syndrome, overweight/obesity, insulin resistance, lipid profile, and hypertension (25, 26, 28–30). These studies reported that PI was inversely related to hypertension, dyslipidemia, obesity, insulin resistance development, and prevalence of metabolic syndrome. However, we are not aware of any study examining PI in metabolically healthy and unhealthy individuals according to Karelis criteria. Therefore, this study aimed to evaluate the association of PI with MHO and MUO phenotypes among Iranian women.

## Materials and methods

### Study subjects

This cross-sectional study is conducted on 228 overweight/obese women from Tehran, Iran, referred to health centers affiliated to the Tehran University of Medical Sciences (TUMS) during 2017–1019. A multistage cluster sampling method was used to recruit the study population. Participants were selected based on the following inclusion criteria: age 18–48 years, body mass index (BMI) ≥25 kg/m^2^, and no ongoing weight loss program or taking weight loss supplements. Participants with CVDs, diabetes, cancer, kidney disease, thyroid disease, acute and chronic diseases, currently menopause, pregnant/lactating, or taking antidiabetic, antihypertensive, lipid-lowering medications, and weight loss supplements were excluded. Subjects who had a daily energy intake lower than 800 or higher than 4,200 kcal were also excluded. All participants signed informed written consent before their entrance to the study. The study protocol was approved by the Ethics Committee of TUMS with the following identification: IR.TUMS.MEDICINE.REC.1399.636.

### Measurement of biochemical parameters

After 10–12 h of fasting, all blood samples were collected into tubes containing 0.1% EDTA. Samples were immediately centrifuged for 10 min at 3,000 rpm, aliquoted, and stored at a temperature of −80°C until analysis. An AutoAnalyzer BT1500 (Selectra 2; Vital Scientific, Spankeren, Netherlands) was used to analyze all the samples. Plasma glucose was measured by the glucose oxidase phenol 4-aminoantipyrine peroxidase (GOD/PAP) method. The glycerol-3-phosphate oxidase phenol 4-aminoantipyrine peroxidase (GPO-PAP) method was used to assess serum TG. Total cholesterol was measured by an endpoint enzymatic method. LDL-C and HDL-C were assayed by the direct method and immunoinhibition. An immunoturbidimetric assay measured the level of hs-CRP as a marker of inflammation. All kits were purchased from Pars Azmoon (Pars Azmoon Inc., Tehran, Iran). Also, the enzyme-linked immunosorbent assay (ELISA) method (Human Insulin ELISA Kit, DRG Pharmaceuticals, GmbH, USA) was used for serum insulin measurement.

### Definition of metabolic health and its components

According to Karelis criteria, metabolic health status was defined as follows: TG ≤1.7 mmol/L, HDL ≥1.3 mmol/L and no treatment, LDL ≤2.6 mmol/L and no treatment, hs-CRP ≤3.0 mg/L, and HOMA-IR ≤2.7. The presence of four or more of the features is a diagnosis of metabolic health. Thus, according to metabolic health, participants are classified into two groups, namely, MHO and MUO (18).

### HOMA-IR calculation

We calculated insulin resistance using HOMA-IR as the following formula: fasting insulin (mUI/L) × fasting glucose (mmol/L)/22.5 (31).

### Assessment of dietary intake and phytochemical index calculation

Dietary intake was examined using a validated 147-item semi-quantitative Food Frequency Questionnaire (FFQ) (32). The FFQ contains a list of foods commonly consumed by Iranians in standard serving sizes. In face-to-face interviews, participants were asked to report their average frequency of food intakes. Finally, we converted the daily intake of all food items to grams per day using household measures.

Phytochemical index was calculated based on the method developed by McCarty {PI = [daily energy derived from phytochemical-rich foods (kcal)/total daily energy intake (kcal)] × 100} (27). This index included phytochemical-rich foods such as whole grains, fruits and vegetables, nuts and seeds, legumes, soy products, and olive and olive oil. The potato was not included due to its low phytochemical content, and it is often consumed as a starch component rather than a vegetable. Natural fruit and vegetable juices and tomato sauce were considered in PI due to their high phytochemical content. We also added tea and coffee as rich sources of phytochemicals. Phenolic compounds, including flavonoids, phenolic acids, hydroxycinnamic acids, lignans, tyrosol esters, stilbenoids, isoprenoids, and organosulfur compounds (allyl sulfurs, isothiocyanate) are taken into account in PI.

### Body composition analysis

A multi-frequency bioelectrical impedance analyzer (BIA), InBody 770 Scanner (Inbody Co., Seoul, South Korea), was used for body composition assessment. Fat-free mass (FFM), fat mass (FM), and waist-to-hip ratio (WHR) were recorded by this analyzer. As the manufacturer’s instructions required, participants had to take their shoes, coats, and sweaters off, stand on the balance scale, and hold the handles of the machine. In addition, subjects were required to abstain from intense exercise and intake of a meal or excessive fluid before the analysis. Body composition analysis was conducted in a fasting condition in the morning after emptying the bladder (33).

### Assessment of other variables

Information on age, education (primary/secondary/university), marriage (single/married), smoking status (smoker/non-smoker), family history of obesity (yes/no), and occupation (employed/unemployed) was obtained.

In terms of anthropometric measurements, weight was measured to the nearest 100 g using digital scales with minimal clothes, and height was measured to the nearest 0.5 cm by a tape meter mounted on the wall while the subject was standing in a normal position without shoes. BMI was calculated by dividing weight (kg) by height squared (m^2^). Waist circumference (WC) was measured by a tape at the level midway between the lower rib margin and the iliac crest at the end of normal expiration to the nearest 0.5 cm when subjects were standing.

The level of physical activity (PA) was assessed using the International Physical Activity Questionnaire (IPAQ). Participants reported the frequency and duration of severe, moderate, jogging, and sedentary PA during the past 7 days, and the level of PA was expressed as metabolic equivalent hours per week (METs/h/week). METs were classified as low (<600 MET-min/week), moderate (600–3,500 MET-min/week), and vigorous (>3,500 MET-min/week).

### Statistical analysis

Kolmogorov–Smirnov’s test was used to evaluate the normal distribution of the data. One-way analysis of variance (ANOVA) was used to determine differences in continuous variables across tertiles of dietary phytochemical index (DPI). The Chi-square test was applied to examine the distribution of participants in terms of categorical variables across tertiles of DPI. Data on quantitative characteristics were reported as the mean ± SD and data on qualitative characteristics were expressed as a percentage. The differences between the dietary intakes of participants based on the obesity phenotypes were assessed by one-way ANOVA. For evaluating these differences after adjustment of age (continuous), BMI (continuous), PA (low, moderate, and high), and total energy intake (continuous), the analysis of covariance (ANCOVA) test was used. Binary logistic regression was applied to examine the associations of the DPI (independent variable) with the MUO (dependent variable) and components of Karelis criteria (dependent variable). DPI was categorized as tertiles, and MUO and the five components of Karelis Criteria were categorized as binary variables in the models. SPSS software was used (version 24; SPSS Inc., Chicago, IL, USA) for all statistical analyses. *P*-value < 0.05 was considered statistically significant.

## Results

The general characteristics of the 228 study participants across tertiles of DPI are shown in Table 1. Compared to the participants in the lowest tertile, participants in the highest tertile were older (*P* < 0.001), more likely to have lower weight (*P* = 0.03) and height (*P* = 0.004), lower FM (*P* = 0.03), FFM (*P* = 0.02), and more likely to be non-smokers (*P* = 0.01).

**TABLE 1:** General characteristics of study participants by tertiles of DPI.

		Tertiles of DPI			
		
	Total (*n* = 228)	T1 (*n* = 76)	T2 (*n* = 76)	T3 (*n* = 76)	*P*-value^a^
**Demographic variables**					
Age (years)	36.69 ± 9.20	33.51 ± 8.27	37.61 ± 9.22	39.97 ± 9.26	<0.001
Weight (kg)	81.17 ± 12.26	83.24 ± 13.26	81.03 ± 11.74	79.24 ± 11.48	0.03
Height (cm)	161.15 ± 5.88	162.08 ± 5.56	161.57 ± 5.61	159.80 ± 6.25	0.004
**Body composition**					
BMI (kg/m^2^)	31.27 ± 4.30	31.84 ± 4.95	30.95 ± 3.66	31.02 ± 4.16	0.18
FM (kg)	34.73 ± 8.74	36.28 ± 9.93	34.27 ± 7.69	33.63 ± 8.29	0.03
FFM (kg)	46.50 ± 5.66	47.32 ± 5.40	46.69 ± 5.99	45.48 ± 5.47	0.02
WC (cm)	99.59 ± 10.07	101.01 ± 10.72	99.47 ± 9.43	98.28 ± 9.91	0.09
WHR	0.93 ± 0.05	0.93 ± 0.05	0.93 ± 0.05	0.93 ± 0.05	0.42
**Biochemical parameters**					
FBS (mmol/L)	4.86 ± 0.53	4.83 ± 0.49	4.86 ± 0.51	4.87 ± 0.58	0.89
TG (mmol/L)	1.38 ± 0.79	1.33 ± 0.82	1.37 ± 0.78	1.42 ± 0.78	0.76
LDL-C (mmol/L)	2.45 ± 0.62	2.37 ± 0.61	2.48 ± 0.60	2.50 ± 0.65	0.40
HDL-C (mmol/L)	1.21 ± 0.28	1.19 ± 0.25	1.22 ± 0.28	1.20 ± 0.30	0.69
Total-C (mmol/L)	4.78 ± 0.93	4.69 ± 0.99	4.83 ± 0.87	4.82 ± 0.95	0.56
HOMA	3.34 ± 1.28	3.23 ± 1.18	3.30 ± 1.35	3.48 ± 1.30	0.46
**Inflammatory marker**					
hs-CRP (mg/L)	4.31 ± 4.65	4.61 ± 4.51	4.18 ± 4.80	4.19 ± 4.66	0.81
**Qualitative variables**					
**Marital status (%)**					
Married	72.2	65.9	77.7	73.1	0.10
Single	27.8	34.1	22.3	26.9	
Family size (%)					
≤4		86.8	79	87.3	0.12
>4		13.2	21	12.7	
**Education (%)**					
≤Primary	4.6	6.2	3.1	4.6	0.65
Secondary	40.6	37.2	40.8	43.8	
University	54.8	56.6	56.2	51.5	
**Occupation (%)**					
Unemployed	59	63.8	60.5	52.7	0.18
Employed	41	36.2	39.5	47.3	
**Smoking (%)**					
Yes	6.9	12.3	4.6	3.9	0.01
No	93.1	87.7	95.4	96.1	
**Economic status (%)**					
Poor	22.9	18.5	25.4	24.8	0.67
Medium	48.5	52.4	47.6	45.6	
High	28.6	29	27	29.6	
**Weight loss history (%)**					
Yes	54.5	49.6	56.8	57	0.42
No	45.5	50.4	43.2	43	
**Family history of obesity (%)**					
Yes	71	72.1	67.7	73.2	0.60
No	29	27.9	32.3	26.8	
**Physical activity (%)^b^**					
Low	50	56.0	42.9	51.2	0.14
Moderate	45.3	41.3	53.8	40.7	
High	4.7	2.7	3.3	8.1	

DPI, dietary phytochemical index; BMI, body mass index; FM, fat mass; FFM, fat free mass; PBF, percent body fat; WC, waist circumference; WHR, waist to hip ratio; FBS, fasting blood sugar; TG, triglyceride; LDL-C, low-density lipoprotein cholesterol; HDL-C, high-density lipoprotein cholesterol; Total-C, total cholesterol; HOMA, homeostatic model assessment; hs-CRP, high-sensitivity C-reactive protein. *P*-value less than 0.05 was considered significant.

All values are mean ± SD or reported percentage.

^a^*P*-values result from ANOVA for quantitative variables and χ^2^ test for qualitative variables.

^b^Physical activity was classified as low <600 MET-h/week, moderate = 600–3,500 MET-h/week, and high >3,500 MET-h/week.

The dietary intakes of participants according to obesity phenotypes are presented in Table 2. Only cholesterol intake was significantly higher among subjects with MUH status (*P* = 0.02) even after adjusting for total energy intake (*P* = 0.04).

**TABLE 2:** Dietary intake of MHO and MUO participants.

	MHO (*n* = 64)	MUO (*n* = 164)	*P*-value^a^	*P*-value^b^
Total energy (kcal/day)	2,516.37 ± 725.67	2,636.87 ± 751.67	0.27	–
Carbohydrate (g/day)	363.26 ± 124.21	374.42 ± 117.81	0.53	0.28
Protein (g/day)	83.29 ± 25.78	90.13 ± 28.47	0.09	0.19
Fat (g/day)	89.77 ± 27.81	95.35 ± 33.56	0.24	0.62
Total fiber (g/day)	45.35 ± 19.98	44.49 ± 17.82	0.75	0.19
SFA (g/day)	26.23 ± 9.78	28.56 ± 11.66	0.16	0.37
PUFA (g/day)	19.21 ± 7.04	20.27 ± 9.00	0.40	0.72
MUFA (g/day)	29.28 ± 8.82	31.65 ± 11.62	0.14	0.32
Cholesterol (mg/day)	225.83 ± 82.04	259.32 ± 105.13	0.02	0.04
Vitamin A (RAE/day)	725.76 ± 349.76	807.93 ± 435.35	0.18	0.34
Vitamin E (mg/day)	16.31 ± 7.23	17.58 ± 9.22	0.32	0.50
Vitamin C (mg/day)	191.31 ± 124.73	199.48 ± 134.42	0.67	0.88
Calcium (mg/day)	1,115.47 ± 420.35	1,172.18 ± 416.70	0.36	0.81
Magnesium (mg/day)	443.31 ± 143.05	469.90 ± 150.17	0.22	0.58
Potassium (mg/day)	4,216.38 ± 1,632.89	4,416.48 ± 1,546.65	0.39	0.96
Zinc (mg/day)	12.29 ± 4.08	13.26 ± 4.31	0.12	0.25
Whole grains (g/day)	56.86 ± 53.47	63.50 ± 62.17	0.45	0.63
Fruits (g/day)	557.51 ± 398.24	565.57 ± 367.16	0.88	0.59
Vegetables (g/day)	425.25 ± 243.12	455.95 ± 279.76	0.44	0.66
Nuts and seeds (g/day)	15.61 ± 16.91	15.60 ± 17.86	0.99	0.64
Legumes (g/day)	48.81 ± 53.05	46.41 ± 31.25	0.67	0.55
Soy sources (g/day)	4.96 ± 8.14	6.06 ± 9.29	0.41	0.43
Olive and olive oil (g/day)	4.11 ± 6.16	4.88 ± 7.58	0.47	0.53
Tea and coffee (g/day)	740.02 ± 575.90	776.88 ± 909.48	0.76	0.88
Phytochemical index	28.15 ± 12.06	26.23 ± 9.48	0.21	0.49

MHO, metabolic healthy obesity; MUO, metabolic unhealthy obesity; SFA, saturated fatty acids; PUFA, poly unsaturated fatty acids; MUFA, mono unsaturated fatty acids. *P*-value less than 0.05 was considered significant. All values are mean ± SD.

^a^*P*-values result from ANOVA.

^b^*P*-values adjusted for energy intake.

Crude and multivariable-adjusted odd ratios (ORs) with 95% confidence intervals (CIs) for MUH phenotype across tertiles of DPI are provided in Table 3. No significant association was found between MUH phenotype and DPI in the crude model. After adjusting for age, BMI, total energy intake, and PA, those in the highest tertile had lower odds for MUH phenotype (OR: 0.29, 95% CI: 0.11–0.76, *P* = 0.01). After further adjustments for marital status, occupation, education, socioeconomic status, weight loss history, and family size, individuals in the highest tertile of DPI still had 77% lower odds for MUH phenotype compared to the lowest tertile (OR: 0.23, 95% CI: 0.07–0.68, *P* = 0.008).

**TABLE 3:** Multivariate adjusted odds ratios (OR) and 95% confidence intervals (CI) for MUO phenotypes across tertiles of DPI.

MUO phenotype	Tertiles of DPI				
	
	T1 (*n* = 76)	T2 (*n* = 76)		T3 (*n* = 76)	
			
		OR (95% CI)	*P*-value	OR (95% CI)	*P*-value
Model 1^a^	1	1.05 (0.48, 2.28)	0.89	0.57 (0.29, 1.14)	0.11
Model 2^b^	1	0.48 (0.11, 1.98)	0.31	0.29 (0.11, 0.76)	0.01
Model 3^c^	1	0.36 (0.07, 1.81)	0.21	0.23 (0.07, 0.68)	0.008

MUO, metabolically unhealthy obesity; DPI, dietary phytochemical index. Results are presented as OR and 95% CI.

^a^Unadjusted.

^b^Adjusted for age, BMI, total energy intake, and physical activity.

^c^Additionally adjusted for marital status, occupation, education, socioeconomic status, weight loss history, and family size.

Table 4 shows multivariate-adjusted models with 95% CIs for components of Karelis criteria among tertiles of DPI. Compared with the subjects in the lowest tertile of DPI, those in the highest tertile had 58% lower odds of having HOMA-IR (OR: 0.42, 95% CI: 0.18–0.97, *P* = 0.04). This association remained significant after additionally controlling for marital status, occupation, education, socioeconomic status, weight loss history, and family size (OR: 0.29, 95% CI: 0.10–0.79, *P* = 0.01). For TG level, in the fully adjusted model, the participants in the highest tertile of DPI had marginally lower odds for TG concentrations above 1.7 mmol/L (OR: 0.35, 95% CI: 0.12–1.03, *P* = 0.05). No significant association was detected between DPI and other components of Karelis criteria.

**TABLE 4:** Multivariate adjusted odds ratios (OR) and 95% confidence intervals (CI) for Karelis criteria components across tertiles of DPI.

Tertiles of DPI					
	
	T1 (*n* = 76)	T2 (*n* = 76)		T3 (*n* = 76)	
			
		OR (95% CI)	*P*-value	OR (95% CI)	*P*-value
**HOMA-IR**					
Model 1^a^	1	0.69 (0.34, 1.40)	0.31	0.49 (0.25, 0.95)	0.03
Model 2^b^	1	0.72 (0.21, 2.50)	0.61	0.42 (0.18, 0.97)	0.04
Model 3^c^	1	0.43 (0.10, 1.83)	0.25	0.29 (0.10, 0.79)	0.01
**hs-CRP (mg/L)**					
Model 1	1	1.70 (0.89, 3.24)	0.10	1.08 (0.57, 2.04)	0.79
Model 2	1	1.79 (0.54, 5.91)	0.33	0.89 (0.38, 2.06)	0.78
Model 3	1	3.00 (0.77, 11.56)	0.11	1.10 (0.42, 2.88)	0.84
**LDL-C (mmol/L)**					
Model 1	1	0.70 (0.36, 1.35)	0.29	1.03 (0.56, 1.91)	0.90
Model 2	1	1.08 (0.34, 3.43)	0.89	1.12 (0.49, 2.51)	0.78
Model 3	1	0.71 (0.19, 2.61)	0.61	0.95 (0.38, 2.36)	0.91
**HDL-C (mmol/L)**					
Model 1	1	0.84 (0.42, 1.64)	0.61	0.66 (0.35, 1.26)	0.21
Model 2	1	0.53 (0.16, 1.70)	0.28	0.58 (0.25, 1.32)	0.20
Model 3	1	0.40 (0.10, 1.50)	0.17	0.43 (0.17, 1.11)	0.08
**TG (mmol/L)**					
Model 1	1	0.71 (0.34, 1.47)	0.36	0.64 (0.31, 1.31)	0.22
Model 2	1	0.68 (0.19, 2.43)	0.55	0.54 (0.21, 1.37)	0.19
Model 3	1	0.42 (0.10, 1.80)	0.24	0.35 (0.12, 1.03)	0.05

DPI, dietary phytochemical index; HOMA-IR, homeostatic model assessment for insulin resistance; hs-CRP, high-sensitivity C-reactive protein; LDL-C, low-density lipoprotein cholesterol; HDL-C, high-density lipoprotein cholesterol; TG, triglyceride.

^a^Unadjusted.

^b^Adjusted for age, BMI, total energy intake, and physical activity.

^c^Additionally adjusted for marital status, occupation, education, socioeconomic status, weight loss history, and family size.

## Discussion

Little has been understood about the association of DPI, a dietary index that is a simple and practical method for assessing dietary phytochemical intake, with metabolic health status in overweight/obese individuals. Although prior investigations have found an inverse association between DPI and metabolic disorders, including hypertension, dyslipidemia, obesity, insulin resistance, and metabolic syndrome, there is no study investigating the DPI in overweight/obese metabolically healthy and unhealthy individuals according to Karelis criteria. As far as we know, this is the first research project evaluating the relationship between DPI and MHO and MUO phenotypes among overweight/obese Iranian females. Based on our findings, DPI was found to be inversely correlated with MUO phenotype, HOMA-IR, and TG levels even after adjustment for potential confounders, while no association was observed between DPI and serum concentrations of HDL, LDL, and hs-CRP.

In accordance with the obtained results, a longitudinal study performed by Mirmiran et al. (34) concluded that the highest quartile category of DPI was inversely associated with 3-year changes in weight, WC, and body adiposity index among 1,983 Tehranian adults. A recent research project evaluated the relationship between DPI and metabolic syndrome components in the Korean population and proposed that the highest DPI quintile was significantly related to a lower prevalence of metabolic syndrome and its components, including abdominal obesity, hyperglycemia, hypertriglyceridemia, and high blood pressure, although WC and the concentrations of HDL did not alter significantly among DPI quintiles (30). Also, a cross-sectional study by Bahadoran et al. (35) disclosed that higher intakes of phytochemical-rich foods are correlated with 66 and 36% reduced risk of abdominal obesity and hypertriglyceridemia as the main cardiometabolic risk factors in 2,567 Iranian adults aged 19–70 years. In the same study, the mean serum HDL level had increasing trends across the increase of phytochemical intake categories, and also participants in the upper quartile of DPI had a lower weight and WC (35). Furthermore, another cross-sectional study examining the association between DPI and serum concentrations of hs-CRP, a marker of low-grade systemic inflammation released by adipose tissue, found no association between hs-CRP and DPI among 170 premenopausal women, while there were significant associations between hs-CRP and several central obesity indices (36). Moreover, Golzarand et al. (25) reported an inverse association between DPI and TG, TC, and non-HDL cholesterol levels among the Iranian population aged 19–70 years in a Tehran Lipid and Glucose prospective study. In addition, DPI was found to be negatively associated with FBS levels and the 2-h oral glucose tolerance test (OGTT) even after controlling for confounders, including BMI, PA, and dietary intake of energy, fiber, carbohydrate, fat, and protein in a case-control study conducted on 300 Iranian adults (37). The discrepancies between the findings of the mentioned studies may be caused by different nationalities, physiological status, lifestyles, and age groups of participants.

The mechanisms of action of phytochemicals, non-toxic and cost-effective bioactive compounds compared to synthetic alternatives, on obesity and its related indices have been studied by several *in vitro* and *in vivo* models (38). Phytochemicals exert anti-obesogenic activities by targeting various obesity-related pathways and regulatory functions, including inhibition of dietary lipid digestion through attenuating pancreatic lipase activities, which are considered as main enzymes responsible for the digestion and absorption of lipids, inhibition of adipogenesis and differentiation of preadipocytes, stimulation of existing adipocytes apoptosis (38–43). Also, phytochemicals are shown to exert weight-lowering effects by elevating energy expenditure through activating brown adipogenesis, browning of white adipose tissue, and upregulating the expression of thermogenic genes, induction of lipolytic pathways, and appetite modulation through regulating various satiety-related hormonal and neurological signals such as the sympathetic nervous system, dopamine, histamine, and serotonin receptors, and adrenalin, leptin, and ghrelin levels in human bodies (38–43).

Despite several remarkable strengths of the present study, including relatively appropriate sample size, adjustment for numerous probable confounders that could potentially affect the results, and examining the relationship between DPI and metabolic phenotypes of obesity among overweight/obese Iranian females for the first time, our findings should be interpreted in the context of some weaknesses. First, as the most significant limitation, the current study cannot exhibit the causal association between DPI, MHO, and MUO phenotypes based on its cross-sectional nature. Second, there was no information about the duration of obesity that has been suggested to affect participants’ metabolic health status. Third, DPI was computed using calories from consumed food items, and thereby food items containing zero calories, such as spices, which are good sources of phytochemicals, could not be considered. Fourth, we used retrospective dietary data by FFQ, which may cause errors in calculating DPI due to the participants’ recall bias. Finally, racial homogeneity was also a limitation of the current research.

## Conclusion

Dietary phytochemical index was found to be inversely correlated with MUO phenotype, HOMA-IR, and TG levels even after adjustment for potential confounders. Further prospective cohort studies with larger sample sizes are proposed to elucidate the association between DPI and metabolic phenotypes of obesity among overweight/obese subjects.

## Data availability statement

The original contributions presented in this study are included in the article/supplementary material, further inquiries can be directed to the corresponding author.

## Ethics statement

The studies involving human participants were reviewed and approved by the Tehran University of Medical Sciences (TUMS) with the following identification: IR.TUMS.MEDICINE.REC.1400.696. The patients/participants provided their written informed consent to participate in this study.

## Author contributions

SP was responsible for conceptualization. SP and AM analyzed the data. SP, FN, and SN took the lead in writing the manuscript. AM and KM were responsible for review and editing. All authors contributed to the article and approved the submitted version.

## Abbreviations

ANCOVA: analysis of covariance
ANOVA: one-way analysis of variance
BIA: bioelectrical impedance analyzer
BMI: body mass index
CI: confidence interval
CVD: cardiovascular disease
DM: diabetes mellitus
DPI: dietary phytochemical index
ELISA: enzyme-linked immunosorbent assay
FFM: fat-free mass
FM: fat mass
FFQ: Food Frequency Questionnaire
GOD/PAP: glucose oxidase phenol 4-aminoantipyrine peroxidase
GPO/PAP: glycerol-3-phosphate oxidase phenol 4-aminoantipyrine peroxidase
HDL-C: high-density lipoprotein cholesterol
HOMA-IR: homeostatic model assessment for insulin resistance
hs-CRP: high-sensitivity C-reactive protein
IPAQ: International Physical Activity Questionnaire
LDL-C: low-density lipoprotein cholesterol
MET: metabolic equivalent
MHO: metabolically healthy obesity
MUO: metabolically unhealthy obesity
OGTT: oral glucose tolerance test
OR: odds ratio
PA: physical activity
TG: triglyceride
TUMS: Tehran University of Medical Sciences
WC: waist circumference
WHR: waist to hip ratio.
